# More Thumbs Than Rules: Is Rationality an Exaptation?

**DOI:** 10.3389/fpsyg.2022.805743

**Published:** 2022-02-24

**Authors:** Antonio Mastrogiorgio, Teppo Felin, Stuart Kauffman, Mariano Mastrogiorgio

**Affiliations:** ^1^IMT School for Advanced Studies Lucca, Lucca, Italy; ^2^Huntsman School of Business, Utah State University, Logan, UT, United States; ^3^Saïd Business School, University of Oxford, Oxford, United Kingdom; ^4^Institute for Systems Biology (ISB), Seattle, WA, United States; ^5^Department of Strategy, IE University, Segovia, Spain

**Keywords:** exaptation, embodied rationality, bounded rationality, heuristics, neural reuse, spandrels

## Abstract

The literatures on bounded and ecological rationality are built on adaptationism—and its associated modular, cognitivist and computational paradigm—that does not address or explain the evolutionary origins of rationality. We argue that the adaptive mechanisms of evolution are not sufficient for explaining human rationality, and we posit that human rationality presents exaptive origins, where exaptations are traits evolved for other functions or no function at all, and later co-opted for new uses. We propose an embodied reconceptualization of rationality—embodied rationality—based on the reuse of the perception-action system, where many neural processes involved in the control of the sensory-motor system, salient in ancestral environments have been later co-opted to create—by tinkering—high-level reasoning processes, employed in civilized niches.

## Introduction

I counted the panda’s other digits and received an even greater surprise: there were five, not four. Was the “thumb” a separately evolved sixth finger?

S. J. Gould, *The Panda’s Thumb*

In Herbert A. Simon’s view, heuristics are *rules of thumb*—instantiations of bounded rationality—that produce solutions adapted to specific task environments, given limited information, time and cognitive capabilities. This adaptive dimension is part of the very definition of *bounded rationality*, also known as the scissors’ metaphor: “Just as a scissors cannot cut paper without two blades, a theory of thinking and problem solving cannot predict behavior unless it encompasses both an analysis of the structure of task environments and an analysis of the limits of *rational adaptation* to task requirements” (Newell and Simon, 1972, p. 55, emphasis added). The adaptive view of rationality—‘rational adaptation’ in Simon’s own words—has been transposed into contemporary views of heuristics. While Kahneman’s ‘heuristics and biases’ focus on the nature of dis-adaptation, in the sense that heuristics, automatically triggered, do not fit specific task environments (Tversky and Kahneman, 1974; Kahneman et al., 1982), Gigerenzer’s ‘ecological rationality’ emphasizes the fact that fast and frugal heuristics produce satisficing solutions, through ecological correlations (Gigerenzer and Selten, 2002; Gigerenzer and Gaissmaier, 2011).

In this contribution, we criticize the emphasis on the adaptative logic (e.g., Cosmides and Tooby, 2006; Tooby and Cosmides, 2007), arguing that adaptive mechanisms are not a unique or sole explanation for human rationality. Relying on the old but still relevant critique to adaptationism—initially raised by Stephen J. Gould (i.e., Gould and Lewontin, 1979; see also Andrews et al., 2002)—we discuss a fundamental limit of adaptative explanations: the difficulty to make a distinction between contingent utility and reasons for origins. For instance, consider the popular and frequently discussed gaze heuristic (e.g., Gigerenzer and Gray, 2017; Hamlin, 2017; Höfer et al., 2018), which is used to track the motion of a moving goal by keeping constant the angle between a catcher and the goal. The fact that a soccer player uses the gaze heuristic to catch a ball (contingent utility) tells us nothing about how such a heuristic came into being in the first place (reasons for origins). We of course realize that the gaze heuristic did not come into being for playing soccer, since it was present thousands of years before soccer was invented and other species also use it (in particular it is heavily used by predators to catch prey). In short, the idea that heuristics are effective decision rules for contingent task environments does not strictly explain their origins.

We argue that exaptive mechanisms are fundamental for explaining the origins of rationality. While adaptations are traits gradually evolved *via* natural selection in order to meet pre-existing functions, *exaptations* are traits evolved for other functions, or no function at all, and later co-opted for new uses (Gould and Vrba, 1982). We propose an embodied reconceptualization of rationality—so-called, *embodied rationality* (e.g., Mastrogiorgio and Petracca, 2012, 2015, 2016)—based on (non-adaptive but) exaptive evolutionary mechanisms. In particular, we amend and reconceptualize bounded and ecological rationality, by discussing the reuse of the perception-action system: many neural processes involved in the control of the sensory-motor system, which were salient in ancestral environments, have been later co-opted to shape—*via* tinkering—high-level cognitive faculties employed in civilized niches.

## Rationality and Adaptation

Evolutionary explanations are elegant from the point of view of Occam’s Razor: by identifying some criteria to explain the factual diversity of nature, evolutionary theories aim to establish theoretically plausible solutions to the problem of origins. In its general definition, also known under the general label of Universal Darwinism, evolution is instantiated by the processes of variation, selection and retention—processes that account for the diversity that composes natural and cultural systems (cf. Campbell, 1960; Lewontin, 1970; Dawkins, 1983; Hodgson, 2005).

The program of research on bounded rationality, started by Herbert A. Simon, owes much to evolutionary frameworks, as it is argued that human behaviors must be studied with respect to specific task environments. Simon emphasizes that minds are adapted to real-world environments and must be evaluated in terms of their adequacy to specific environmental instances: in the scissors’ argument (summarized above), cognition and environment are two cutting blades that make sense precisely because they are conjointly defined in a unitary analytical framework (Simon, 1956, 1990; Newell and Simon, 1972).

Modern transpositions of bounded rationality are sympathetic to this adaptive, evolutionary framework. On the one side, Kahneman’s *heuristics and biases* are based on the evidence that specific heuristics, which are automatically triggered, violate specific rules of logic and probability so as to produce biased judgments (Tversky and Kahneman, 1974; Kahneman et al., 1982). On the other side, Gigerenzer’s *ecological rationality* relies on the idea that fast and frugal heuristics, by exploiting ecological correlations, provide satisficing solutions to specific task environments (Gigerenzer and Goldstein, 1996; Gigerenzer and Gaissmaier, 2011), where ‘satisficing’ is a well-known neologism coined by Simon, given by the combination of ‘satisfy’ and ‘suffice.’

These two colliding research programs are at the center of so-called ‘rationality wars’ (see Samuels et al., 2004 for a discussion). In fact, Gigerenzer criticizes Kahneman’s heuristics as being “vague, undefined, and unspecified with respect both to the antecedent conditions that elicit (or suppress) them and also to the cognitive processes that underlie them” (Gigerenzer, 1996, p. 592). Generally speaking, Gigerenzer criticizes the incorrectness of deducing a positive framework of rationality by relying on the experimental evidence built upon the normative benchmarks based on general rules of logic and probability calculus. This critique (Gigerenzer and Murray, 1987; Gigerenzer, 1991) created a dialectical interaction and debate (see the replies of Gigerenzer, 1996; Kahneman and Tversky, 1996). In such rationality wars, while Gigerenzer’s view remains “panglossian,” in the sense that fast and frugal heuristics generate satisficing solutions, Kahneman’s view is “meliorist,” in the sense that heuristics are sources of cognitive errors and biases and produce misfits with respect to specific normative requirements.

### Inside the Adaptive Toolbox

Fast and frugal heuristics generate satisficing outcomes to the extent that they fit the specific structure of the task environment. This idea is a pillar of ecological rationality, which investigates “in which environments a given strategy is better than other strategies” (Gigerenzer and Gaissmaier, 2011). The research program on ecological rationality, developed by Gigerenzer and Gaissmaier (2011), emphasizes that heuristics compose an *adaptive toolbox*, where human behavior is described by a series of “cognitive heuristics, their building blocks (e.g., rules for search, stopping, and decision), and the core capacities (e.g., recognition memory) they exploit.” In Gigerenzer (2008, p. 20) own words: “The adaptive toolbox is a Darwinian-inspired theory that conceives the mind as a modular system that is composed of heuristics, their building blocks, and evolved capacities.”

Using an adaptive framework to explain the nature of heuristics would imply that heuristics are (casual) variations selected by the environment because of their comparatively better fitness, which are then retained. For instance, let us consider the evidence that some fast and frugal heuristics predict heart attack in a manner that is comparable with complex, effortful (and slow) medical procedures (Todd and Gigerenzer, 2000; Marewski and Gigerenzer, 2012). If we adopt a strict, adaptationist, evolutionary framework—based on variation, selection and retention—, we should hypothesize that these heuristics are the result of a gradual refinement of older ones, that have been selectively retained by the environment. Despite the emphasis on the adaptive toolbox, theoretical inquiries on the role of selection—which is fundamental for adaptation—seem to be overlooked in ecological and bounded rationality, where the selective mechanisms remain underexplored.

Interestingly, the absence of theorizing on the nature of adaptation is not a bug of ecological rationality but a deliberate theoretical choice. On this point, Hutchinson and Gigerenzer (2005; see also Sanabria and Killeen, 2005) clarify that the adaptive view of heuristics is not an argument about their origins. That is, the fact that a heuristic is ecologically rational does not imply that it has been shaped by the forces of selection for that specific task. Interestingly, ecological rationality avoids both trivial adaptive explanation (“just-so stories”) and the necessity of accurate theorizing on the nature of origins: “It thus would be a weak argument […] to find a heuristic that humans use, then search for some environment in which that heuristic works well, and then claim on this basis alone that the heuristic is an adaptation to that environment. The heuristic may work well in that environment, but that need not be the reason why it evolved or even why it has survived” (Hutchinson and Gigerenzer, 2005, p. 109; see also Navarrete and Santamaría, 2011). This clarification, though, looks like an *excusatio non-petita*, where the caveat substitutes the claim for an adaptive toolbox. As the authors add (p. 109): “Ecological rationality might then be useful as a term indicating a more attainable intermediate step on the path to a demonstration of adaptation. There is nevertheless a risk that a demonstration of ecological rationality of a given heuristic in a given environment will mislead someone who uses this evidence alone to infer adaptation.”

The risk of making casual and cursory claims about the evolutionary origins of heuristics is real, but the above clarification is hopelessly insufficient. That is, if we cannot infer adaptation, then why even speak of an *adaptive* toolbox? The authors’ clarification—about the problem of inferring adaptation from ecological rationality of a given heuristics—therefore, raises important issues, which are crucial in the critique of adaptationism.

### Adaptationism at Stake

For decades, S. J. Gould, in his broad program of research, has tried to demonstrate that there is an unjustified, paradigmatic correspondence between the general problem of evolution as originally formulated by Charles Darwin and its transposition into the neo-Darwinian synthesis. According to the dominant paradigm, there are no radical alternatives to ‘adaptationism.’ When scholars in different fields refer to evolutionary theories, they are, implicitly and unwittingly, appealing to a mechanism that they consider necessary and sufficient: adaptation. Therefore, according to Gould—and consistently with Kuhn’s idea of a paradigmatic science—adaptationism signals more a faith in evolutionary theorizing (where the risk of “just-so stories” is always present) than a deep understanding of its related questions. The theoretical mechanisms and implications of adaptationism are a matter that cannot be informally treated in a few words. Generally speaking, as suggested by Gould (and by Darwin himself), the laws of change, more than a nomothetic necessity, should be considered as extrapolations whose instrumental value is the understanding of empirical evidence (Gould and Eldredge, 1977; Gould and Lewontin, 1979; Gould and Vrba, 1982; Gould, 2002, see also Williams, 1966). In particular, according to Gould and Lewontin (1979, p. 581), adaptationism “is based on faith in the power of natural selection as an optimizing agent. It proceeds by breaking an organism into unitary ‘traits’ and proposing an adaptive story for each considered separately. Trade-offs among competing selective demands exert the only brake upon perfection; non-optimality is thereby rendered as a result of adaptation as well”.

Although we acknowledge the disapproval of some scholars (in particular John Maynard Smith and Richard Dawkins) of Gould’s ideas (see Gould, 1997 for a reply), we believe that Gould’s critique of adaptationism matters a great deal for understanding the contemporary state of the art of human rationality, when related with alternatives to adaptive processes. Gould criticizes adaptationism for its unwillingness to consider alternatives to adaptive processes. According to Gould and Vrba (1982, p. 5), there are two meanings of the word ‘adaptation’: “the first is consistent with the vernacular usage […]: a feature is an adaptation only if it was built by natural selection for the function it now performs. The second defines adaptation in a static or immediate way as any feature that enhances current fitness regardless of its historical origin.” Adaptationism fails because of its impossibility to make a distinction between current utility and reasons of origin. Importantly, the fact that a trait satisfies (more or less effectively) a particular function cannot strictly be an explanation of its origins.

According to this critique, adaptation often presumes an unjustified teleological perspective, where things (like biological traits, cognitive faculties or, in our case, rationality) are explained in terms of their final causes, and implicitly represent the best state of the world precisely in virtue of their existence. This part of Gould’s critique—according to which adaptationism is Panglossian—calls to the mind Voltaire’s novel of Candido. Things are made for the best purpose, as suggested by Dr. Pangloss (a character of the novel): “Legs were clearly intended for breeches, and we wear them.” The consideration of the current traits as adaptations often collapses onto the problematic statement that they represent the best status possible in nature, or, quoting again Dr. Pangloss: “Things cannot be other than they are.”

Generally speaking (as we discussed in the previous Section “Inside the Adaptive Toolbox”), both ecological and bounded rationality tend to propose an adaptive view of heuristics, where heuristics are instrumental to the evolution of complex cultures (for instance, Finnish mushroom foragers have learned some rules of thumb to deal with poisonous species, Kaaronen, 2020). Oddly, bounded and ecological rationality overlook the role of selective processes, which might play a fundamental role in the origins of heuristics. This somehow represents an inconsistency, since selection is a crucial mechanism of adaptation. The reasons for this apparent inconsistency are essential and find an adequate answer in Gould’s critique of adaptationism. Bounded and ecological rationality implicitly assume that adaptive mechanisms require *contingent utility*: that is, if a heuristic works in a specific task environment—in the sense that it produces a satisficing outcome—, then it is adapted or ‘ecologically rational,’ in the words of Gerd Gigerenzer. However, the comparatively better performance of a heuristic with respect to alternatives is not, strictly speaking, an explanation of its origins. As we will explain in the next sections, the distinction between ‘contingent utility’ and ‘reasons for origins’ thus represents a fundamental argument that can cast new light on non-adaptive mechanisms at the origins of human rationality.

## The Exaptive Origins of Rationality

The use of adaptive explanations to understand the nature of human rationality represents a significant innovation with respect to standard economic theories, which assume that economic agents are optimizers, thus possessing unconstrained knowledge, time and computational power. Arguments based on bounded rationality approach reasoning processes not in absolute terms (i.e., in terms of logic and probability rules that abstract from a specific environment), but as a matter of domain-specific adequacy of reasoning processes to specific environmental instances. However, such an adaptive framework suffers from the same limits that characterize adaptationist explanations in evolutionary theory. As we discussed in Section “Rationality and Adaptation,” we cannot, strictly speaking, explain the origins of heuristics by considering their contingent utility.

Insights for understanding the exaptive nature of rationality can be drawn from a more pluralistic view on evolution, often called ‘extended evolutionary synthesis’ (Gould, 1982; Pigliucci and Müller, 2010; Laland et al., 2015), which also includes the theory of punctuated equilibrium (Eldredge and Gould, 1972). Far from being an alternative to the Darwinian framework—a paradigm-shift, *stricto sensu*—the extended evolutionary synthesis calls for an exegesis of the original ideas of Darwin, who argued that selection, despite being central, is non-exclusive. As put by Darwin: “it has been stated that I attribute the modification of species exclusively to natural selection […] I am convinced that natural selection has been the main, but not the exclusive means of modification” (Darwin, 1872). In other words, the extended evolutionary synthesis claims the right to adopt a pluralistic approach to evolutionary explanations, challenging the limitations of the modern synthesis.

An extended taxonomy of fitness suggests that we should include exaptive mechanisms, along with adaptive ones, in evolutionary frameworks (Gould, 2002). Such an extension does not deny that adaptation is a fundamental mechanism of evolution, as it just places emphasis on the fact that mutations can occur only ‘given a structure’: current structures, *de facto*, constrain the possibility of evolution more than selective mechanisms effectively do. As suggested by Gould and Lewontin (1979, p. 581; see also Andrews et al., 2002), “the constraints themselves become more interesting and more important in delimiting pathways of change than the selective force that may mediate change when it occurs.” According to this perspective, evolution is more a matter of possibilities and constraints than a matter of optimal fit to a given environment. In particular, Gould and Lewontin, in their manifesto—The spandrels of San Marco and the Panglossian paradigm: a critique of the adaptationist program—propose a structuralist view of evolution, that separates function from structure, by focusing on the so-called *spandrels*. Spandrels are architectural features, given by the roughly triangular spaces between the top of an arch and the ceiling (like the ones in the Basilica of San Marco Basilica in Venice). Evolutionary speaking, spandrels are phenotypic traits that arose as a by-product of evolution of some other traits rather than a product of adaptive selection.

Under such a framework, exaptive mechanisms are complementary to adaptive ones. The basic idea of an extended taxonomy is that a trait contributing to fitness is, simply speaking, an ‘aptation’, until we have sufficient evidence to consider it an ad-aptation or an ex-aptation (see Pievani and Serrelli, 2011). The word ‘ex-aptation’, which etymologically contrasts with ‘ad-aptation’ (where ‘*ex*’ is the Latin correspondent of ‘from’ and ‘*ad*’ is the Latin correspondent of ‘to’), refers to the process through which existing traits, originally developed for a certain use, are employed for uses that are entirely different from the original one (see Table 1). That is, *exaptations* are “characters, evolved for other usage (or no function at all) and later “co-opted” for their current role” (Gould and Vrba, 1982, p. 6). That is, exaptations can be based on either a (1) functional shift or a (2) cooptation of a non-aptation. A common example of functional shift are the feathers of birds, first evolved for thermal regulation, then co-opted for flight. An example of a cooptation of a non-aptation are the sutures in the skulls of young mammals, a byproduct of the laws of growth and then co-opted for aiding parturition. Hence, characteristics or traits must be evaluated not only in terms of effectiveness to a pre-stated *function* but with respect to the affordable *effects* they can produce because of their specific morphological features.

**TABLE 1:** A typology of fitness.

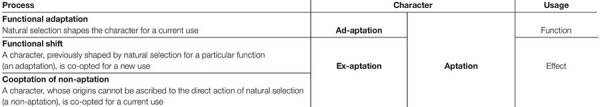

*Adapted from Gould and Vrba (1982).*

Now, in order to understand the exaptive origins of heuristics, let us consider, again, the gaze heuristic (defined in Section “Introduction,” see Raab and Gigerenzer, 2005; Hamlin, 2017; Höfer et al., 2018), which requires an ongoing adjustment between gaze and movement, linking perception and action, to accomplish tasks such as catching a prey or a ball. We can, speculatively, consider the gaze heuristic as a:

(i) *Cooptation from non-aptation.* The morphological traits of the human body strongly constrain the heuristics that can be generated. We can easily realize that the link between gaze and movement, which requires a well-developed sensory-motor integration between perception and action, is a necessary condition for the come-into-being of the gaze heuristic. This consideration seems trivial, unless we consider that this integration is not obvious, since we can reasonably hypothesize that, for some species, such integration is not well developed. For such species the gaze heuristic would not be an affordable option (e.g., we can, speculatively, hypothesize that sloths do not use the gaze heuristic). If we consider this integration as a non-aptation, as we do not assume that this integration is the product of natural selection, we can hypothesize that this integration has been co-opted to create a mechanism—the gaze heuristic—that presents specific advantages (i.e., catching prey) in specific ancestral environments.
(ii) *Functional shift*. If we hypothesize that the gaze heuristic was selected in ancestral environments to be incrementally refined as a successful strategy for catching prey, we can easily realize that such a heuristic has been further exapted, as it readily admits a number of different ‘applications’ in non-ancestral environments. Far from being domain-specific, the gaze heuristic is successfully applied in pedestrian behavior, sailing, landing an airplane, kicking a ball, and so on. Such functional shifts require an isomorphism in the structure of the environment, where the spatial analogies are meaningful. However, analogical correspondence is not necessary. As in the case of feathers of birds, first evolved for thermal regulation then used for flight, we do not always need an isomorphism.

### Cross-Level Mechanisms: Exapting the Perception-Action System

Exaptive mechanisms are not bounded to a specific level but operate, upward and downward, in the hierarchy—genes, organisms and species—of biological systems (Vrba and Gould, 1986). Generally speaking, an adaptation at one level could become an exaptation at another level, as the unit of selection pertains to all the different levels of biological organization (Lewontin, 1970). Interestingly, cross-level mechanisms could represent an interface between nature and culture (Uchiyama et al., 2020), where genetic adaptations could be culturally exapted. For instance, skin pigmentation—a genetic adaptation (a protection against UV radiation)—has been culturally exapted to become a signal of socio-economic status, because over centuries light pigmentation was a signal of high socio-economic status and prestige. But during the 60s, tanning started to be associated with wealth, leisure and prosperity. Gaze heuristics, in this regard, is quite representative, as—we suppose—it first glimmered as a cooptation of non-aption, and then, after the refinements of selective adaptation, it was further exapted in cultural environments through functional shifts.

Here we embrace the hypothesis that such cross-level mechanisms connote the exaptation of the perception-action system, where the sensory-motor devices and their mechanism admit very different applications—high-level cognitive faculties involved in reasoning processes—with respect to the ones for which they evolved through functional adaptation. In particular, an unconstrained view of the unit of selection, along with the presence of exaptations at different levels, allows for the integration of evolutionary and task-relevant timescales. We propose to conceptualize heuristics not only in terms of their original adaptive function (many of them arose in hunter-gatherer environments), but also with respect to their contingent effects, based on exaptive mechanisms, in civilized niches (For instance, the gaze heuristic presents quite different applications such as landing a plane or catching the ball, in civilized environments).

The morphological features of organisms—*bauplan* or *baupläne* (plural)—constrain which heuristics can be developed in the first place, before they are subject to any selective pressures. Hence, human rationality can be considered an ‘adjacent possible’ (cf. Kauffman, 2000) since the enabling constraints, exerted by the sensory-motor system in delimiting the evolution of higher cognitive faculties, are probably more important than the selective forces in mediating changes, once they’ve occurred. (Notice that we use the expression ‘sensory-motor system’ in the singular form as an exemplification, but we are aware that there is a multitude of sensory-motor systems.) Our perspective shifts the emphasis from selective processes to the mechanisms of variation, where the randomness of mutations deserves a clarification. That is, mutations are not equiprobable, as extant biological structures delimit the degrees of freedom and the probabilistic topology of evolutionary possibilities.

The exploitation of exaptive arguments in psychology is not new. However, they are not the orthodoxy, since exaptive arguments have been strongly criticized and ostracized (see Buss et al., 1998 for a critical review of exaptation in psychology). In spite the dialectical role of exaptive arguments in evolutionary psychology (Gould, 1991), we cannot avoid mentioning the heated debate where Darwinian fundamentalism was opposed to a more pluralistic approach (see Dennett, 1997; Gould, 1997; Kalant et al., 1997). We believe and, in this regard, we agree with a number of scholars—though this is not the place for this discussion—that a significant part of the arguments for ostracisms are not persuasive and can be readily neutralized with a philological exegesis of S. J. Gould’s arguments (Lloyd and Gould, 2017).

### Neural Reuse

A fundamental evolutionary argument for an embodied reconceptualization of rationality proposed here consists in the general hypothesis that the brain might be seen as structured into layers, each one presenting a distinctive evolutionary dimension. Neural circuits evolved for specific uses (ad-aptations) or no uses at all (non-aptations) can be co-opted for novel uses, while retaining their original function. This view has gained importance in the last decade, under the general hypothesis that evolutionarily-speaking older brain areas are recruited to support different and relatively novel cognitive functions: recent layers (dedicated to high-level cognitive processes) exploit the ‘lower’ layers, dedicated to the sensory-motor control (Anderson, 2007a).

This perspective can captured under the notion of *neural reuse*, an umbrella term for a heterogenous group of overlapping theories (see Rathkopf, 2021) sharing this view of the brain. This group includes the ‘Massive Redeployment Hypothesis’ (Anderson, 2007a,^2010^), ‘neuronal recycling’ (Dehaene and Cohen, 2007), ‘neural exploitation’ (Gallese, 2008), ‘neural repurposing’ (Parkinson and Wheatley, 2015), ‘cognitive recycling’ (Barack, 2017) and ‘neural exaptation’ (Chapman et al., 2017).

Neural reuse, in particular in its version known as the ‘Massive Redeployment Hypothesis’, represents an alternative to both strict brain localization of cognitive functions—the orthodox position on the functional topography of the brain—and holistic approaches to the brain (Anderson, 2007b,^2014^, see also Favela, 2021 for a critical discussion of the evolutionary foundations of neural reuse). This novel view of the brain, with respect to the orthodox perspective—where specific cognitive functions are strictly localized in specific, non-overlapping areas—builds upon a methodological consideration: subtractive methods used in brain imaging are problematic when it comes to interpreting data and the conclusions these interpretations support. As put by Anderson (2007b, p. 148), “while difference images can show areas that participate in one task and not another, they cannot show that the area is limited to that task.” Indeed, the evidence is that “there are very few specialists in the brain, supporting only tasks from a single task category such as semantics or visual perception. Most regions of the brain are active during multiple tasks in different task categories” (Anderson, 2016, p. 2; see also Anderson et al., 2013). There is of course ongoing debate about whether specific areas of the brain can be mapped onto specific cognitive processes (e.g., Poldrack, 2006). But, the Massive Redeployment Hypothesis posits that a typical cognitive function involves more than one brain area, and each brain area may be redeployed in support of other cognitive functions, according to a three-tier architecture characterized by many-to-many relationship between each level (brain area, component function, and functional complex). Again, in the words of Anderson (2007b, p. 163), we expect “each functional complex to have more than one component, each of which in turn will involve more than one area; likewise, we should expect areas to be members of more than one component, and components to be members of more than one functional complex, and we should not expect that such cross-participation will respect traditional functional-anatomical boundaries.”

Evidence of the Massive Redeployment Hypothesis (where ‘massive’ indicates that redeployment is the norm in the brain), such as the sensorimotor coding in working memory or motor simulations in language understanding (see Anderson, 2006, 2007b), thus represents an argument in favor of cross-level exaptive mechanisms, where lower brain areas participate in higher cognitive processes. Such a thesis is also corroborated by the evidence that brain lesions can produce deficits across multiple domains, thus representing a solid counterargument to the modularity thesis (Prinz, 2006).

We believe that the Massive Redeployment Hypothesis can serve as a building block for an embodied foundation of human rationality. Indeed, the Massive Redeployment Hypothesis, by its own tenets, sheds light not only on the origins of novel, high-level, cognitive functions (in which a given circuit was redeployed), but also on the older functions and structures from which it originates (Anderson, 2008), or, put differently, it is theoretically salient on both sides.

#### The Case of Fingers in Numerical Cognition

The anecdotal case of the aforementioned exapted gaze heuristic (discussed in Section “The Exaptive Origins of Rationality”)—both as functional shift and cooptation of a non-aptation—is relatively trivial, since the gaze heuristic by definition remains bounded to perception-action mechanisms. But what about the exaptive origins of higher cognitive processes involved in human rationality? In order to shed light on this point, consider the case of fingers in numerical cognition.

A crucial argument that is central in the ‘rationality wars’ (discussed in Section “Rationality and Adaptation”) is related to the nature of numerical cognition, and in particular to frequency formats (Gigerenzer and Hoffrage, 1995). Gigerenzer (1996) and his colleagues have tested models that predict when frequency judgments are valid and when they are not. According to Gigerenzer, some cognitive biases—for example, the well-known conjunction fallacy (e.g., Tversky and Kahneman, 1983)—can be neutralized if probability information, in specific task environments, is given in absolute frequencies, not percentages (the so-called ‘natural frequency hypothesis’: Hertwig and Gigerenzer, 1999; see Amitani, 2015 for a discussion). What seems to be interesting in this ‘frequency battle’ is that both perspectives are quite silent on the nature of numerical cognition and, in particular, on the evidence that numerical processing is significantly embodied.

An important tradition of research over the last two decades highlights that numerical processing is constitutively dependent on the sensory-motor system: “Adults can be said to rely on an abstract representation of number if their behavior depends only on the size of the numbers involved, not on the specific […] means of denoting them” (Dehaene et al., 1998, p. 356). Numerical processing depends on the surface format, as magnitudes are denoted by employing numeral systems (e.g., decimal system, binary system, graphical systems, etc.) and their respective notations (Arabic notation, Roman notation, etc.) (for an articulate view on this debate, see Cohen Kadosh and Walsh, 2009). For instance, ‘four’ can be expressed as ‘OOOO, ‘ ‘4’ in the decimal numeral system, ‘100’ in the binary numeral system, ‘IV’ in the Roman numeral system. What we call numbers are actually numerals, namely artifacts that humans manipulate in order to perform computations. As suggested by Lakoff and Núñez (2000, p. 86): “when we learn procedures for adding, subtracting, multiplying, and dividing, we are learning algorithms for manipulating symbols-numerals, not numbers.” With a specific reference to heuristics, Mastrogiorgio and Petracca (2014) show that what selectively activates automatic or deliberate systems, in the well-known ‘bat and ball’ problem (Frederick, 2005), are the specific numerals involved in the task. This type of evidence corroborates the idea that heuristics processing of magnitudes is not neutral to the format of the task. Actually, the format of the task is part of the task and is precisely what enables a specific type of solving process (Mastrogiorgio, 2015).

The embodied, non-abstract view of numerical cognition represents an argument that is just as crucial to the rationality debate as it is overlooked. The perception-action system is not an accessory of mathematical cognition, but it is precisely the embodied substrate exapted for the emergence of higher-level faculties. The embodied dimension of numerical abilities, where numerical processing is grounded on the perception-action systems, represents a fundamental argument to consider such abilities as exaptations of the perception-action system. Indeed, logical and mathematical abilities—which are considered as pillars of rationality—are actually embodied and require neural reuse. Walsh (2003) highlights that the sensory-motor system provides a common metric for different types of magnitudes (space, time, and numbers). The idea is consistent with the hypothesis that the manipulation of sizes is embodied (see Bueti and Walsh, 2009; Ranzini et al., 2011), where reasoning processes are grounded on the perception-action system (Gallese and Lakoff, 2005). In particular, numerical processing involves gestures of hand (e.g., Chiou et al., 2012) and fingers (e.g., Sato et al., 2007).

More specifically, finger gnosis represents an alternative mechanism that is contrary to the general hypothesis that counting on fingers is the main mechanism on which numerical processing is grounded. *Finger gnosis*—which is the ability to distinguish which finger has been lightly touched without relying on the visual feedback—enables, *via* neural reuse, numerical processing. Indeed, finger gnosis represents the embodied register for storing the numbers to be manipulated. Such a finger register is co-opted for numerical processing, and potentially for all those functions that can exploit such type of biological structure (see Penner-Wilger and Anderson, 2008). Finger gnosis is a good predictor of children’ mathematical performance, but the same cannot be said for the generalist idea of using fingers to count. As discussed by Anderson (2008, p. 432) “children with developmental coordination disorder (DCD) have poor finger agility, but most have preserved finger gnosis, and do not generally evidence significant mathematical difficulties” (see also Cermak and Larkin, 2001).

## More Thumbs Than Rules

Bounded rationality flourished in the *cognitivist paradigm*, according to which reasoning processes are conceived as computational rules to manipulate symbolic representations of the environment (cf. Newell and Simon, 1972). Accordingly, in Gigerenzer’s ecological rationality human behavior can be described by a number of “cognitive heuristics” and “their building blocks (e.g., *rules* for search, stopping, decision)…” (Gigerenzer and Gaissmaier, 2011, p. 456 emphasis added). The cognitivist paradigms focus on abstract computation is further evident in the emphasis that is placed on humans as “intuitive statisticians” (Gigerenzer and Murray, 2015). That is, heuristics are said to be based on various computational and statistical techniques including statistical sampling, threshold and signal detection, just-noticeable-differences, and Bayesian inference (e.g., Gigerenzer and Hoffrage, 1995; Dhami et al., 2004; Karelaia and Hogarth, 2008; Hertwig and Pleskac, 2010; Luan et al., 2014; Gigerenzer, 2019).

However, what is missing in much of this cognitivist focus is that since the 90s, cognitive sciences have been subject to a radical renovation that challenges the assumption of the cognitivist paradigm, through the general hypothesis of a constitutive dependence of cognition on the traits of the human body. Far from being a unitary epistemic attempt, such renovation—known under the general label of *embodied cognition*—includes a pluralism of approaches, differing in their epistemic, theoretical and methodological dimensions (for an overview see Wilson, 2002; Calvo and Gomila, 2008; Clark, 2008; Kiverstein and Clark, 2009; Newen et al., 2018). The flourishing field of embodied cognition—which places a novel emphasis on the sensory-motor system as a constitutive component of cognitive processes—represents a fresh theoretical viewpoint for a reconceptualization of bounded and ecological rationality. This reconceptualization—so-called, *embodied rationality*—considers human rationality as an embodied phenomenon (Spellman and Schnall, 2009; Mastrogiorgio, 2011; Mastrogiorgio and Petracca, 2012, 2015, 2016; Gallagher, 2018; Gallese et al., 2021; Petracca, 2021). Embodied rationality, by endorsing an anti-Cognitivist stance, is critical toward the idea that human rationality is based on symbolic manipulations of a represented environment and, *de facto*, rejects the cognitivist pillar of a cognition implementable on artificial architectures (i.e., the so-called ‘physical symbol system hypothesis,’ Newell and Simon, 1976).

As we discussed in the previous Section “The Exaptive Origins of Rationality,” the exaptation of perception-action systems represents a fundamental mechanism for the come-into-being of higher-level cognitive faculties involved in reasoning processes. Exaptive mechanisms are able to cast light on how the morphological traits of the human body are co-opted to give rise to cognitive mechanisms. Embodied rationality—by claiming an embodied view of cognition and by endorsing (as we propose here) exaptive evolutionary mechanisms— radically challenges the two pillars of evolutionary psychology and ecological rationality: computationalism (i.e., cognitivism) and adaptationism.

### Putting Embodied Rationality Into the Evolutionary Psychology Debate

The idea that biased minds make better inferences is a central argument of ecological rationality (Gigerenzer and Brighton, 2009), antithetical to Kahneman#x2019;s (2011) focus on cognitive biases, which are considered sources of systematic irrationality. Evolutionary psychology, in its foundational principles, is sympathetic with this argument as it views such ‘biasedness’ as a constitutive property of the mind, ascribed to a natural endowment. With reference to the frequency format (discussed in Section “The Case of Fingers in Numerical Cognition”), Tooby and Cosmides (2005, p. 23) endorse natural frequencies (i.e., absolute frequency), admitting that “Giving people probability information in the form of absolute frequencies—an ecologically valid format for hunter-gatherers—reveals the presence of mechanisms that generate sound Bayesian inferences.” Cosmides and Tooby’s (2013) emphasis on the environment of evolutionary adaptedness argues that the modules of the functional architecture of the mind were the product of selective pressure in hunter-gatherer environments but not in civilized ones. Generally speaking, a distinctive mark of evolutionary psychology lies in the general hypothesis that the psychological architecture consists of reasoning and learning processes that are *not* general-purpose, content-independent and somehow equipotential (Tooby and Cosmides, 1992; Pinker, 2002). Mind is not a *tabula rasa* (blank-slate) and organisms come “factory-equipped” with evolutionary endowments allowing specific reasoning and learning processes, which are salient in the respective environments.

We agree with such principles of evolutionary psychology to the extent that we here propose an embodied theory of human rationality that takes into account specific evolutionary mechanisms. Nevertheless, we think that adaptationism and computationalism—both central in evolutionary psychology and the associated literature on ecological rationality—are quite problematic for a theory of embodied rationality, which calls for an embodied view of cognition and asks us to carefully consider (as we propose here) non-adaptive evolutionary mechanisms. Moreover, we cannot avoid noticing that ecological rationality is still—in our opinion—far too anchored on the adaptationism and computationalism of evolutionary psychology, as it deliberately relies on the adaptive arguments of the cognitivist framework (where heuristics are computationally modeled as search and stopping rules for information processing). In the next sections, we propose five arguments that we deem constitutive of embodied rationality and that are dialectically critical against some of the foundational tenets of adaptationist approaches to evolutionary psychology.

#### Tinkering and Rationality

According to Tooby and Cosmides (2005, p. 16), “the brain was designed by natural selection to be a computer. Therefore, if you want to describe its operation in a way that captures its evolved function, you need to think of it as composed of programs that process information.” (This strong focus on information processing readily carries into current work within ecological rationality as well – for example, see Gigerenzer, 2019.) It’s important to point out that Tooby and Cosmides (2005) foundational claim—that the brain is computational and that it is composed of programs of information processing—is extremely provocative and strong, at least for endorsers of embodied cognition. Although they add that “its programs were designed not by an engineer, but by natural selection” (p. 16), we cannot but be puzzled by the juxtaposition of ‘design’ and ‘natural selection,’ also considering that the statement seems to denote a teleological perspective, where things are “designed … to be” and Pittendrigh’s (1958) teleonomy/teleology distinction is not declaratively assumed.

A well-known alternative to unitary-design arguments (i.e., the design of a computer, in Cosmides and Tooby’s words) is that of *tinkering* (Jacob, 1977), according to which the outcomes of evolution do not resemble perfect products of engineering but the ones of a tinkerer, “who does not know exactly what he is going to produce but uses whatever he finds around him” (p. 1163). As further put by Solé et al. (2002, p. 21), “evolution is limited by the constraints present at all levels of biological organization as well as by historical circumstances.” Evolution does not somehow produce novelty from scratch but works on what already exists, as natural selection is strongly dependent on historical contingencies. With reference to the brain, Jacob (1977) adds: “Although our brain represents the main adaptive feature of our species, what it is adapted to is not clear at all” (p. 1166), arguing that the human brain is the product of evolutionary tinkering: “brain development in mammals was not as integrated process as, for instance, the transformation of a leg into a wing. The human brain was formed by superposition of new of new structures on old ones” (p. 1166). This idea critically departs from Cosmides and Tooby’s view (though it is three decades antecedent), in the sense that the brain is not only far from being a perfect device, but also a layered structure where new structures of the neocortex were awkwardly superposed on the old ones through a tinkering process resembling the process of “adding a jet engine to an old horse cart” (p. 1166).

In this contribution, we are sympathetic with tinkering as we hypothesize that human rationality presents exaptive origins, where the ancestral sensory-motor system represents a structure that enables and constrains the emergence of specific reasoning processes, through neural reuse. From this perspective, rationality—far from being the apex of evolutionary processes—is essentially an accidental byproduct whose specificities are evolutionary constrained by contingency. Rationality plausibly resembles a “kluge” (see Marcus, 2008) and can be considered as an ‘adjacent possible’ (Kauffman, 2000), where the historical contingency defines the specificities for a (re)use of bodily structures.

#### Rationality, Out of the Vat

According to Tooby and Cosmides (2005, p. 17): “Individual behavior is generated by this evolved computer, in response to information that it extracts from the internal and external environment.” The emphasis on information processing and the declared computationalism of Cosmides and Tooby is antithetical to the anti-cognitivist arguments of embodied cognition scholars.

Critical precursors of this tension can be found in a quite-known special issue (led by Herbert A. Simon and his colleague Alonso Vera on *Cognitive Science* in the early 90s) on the nature of situated cognition. Traditionally, situated cognition emphasizes that humans think on the fly—through an extemporaneous interaction with the environmental contingencies—, rather than storing and retrieving conceptual knowledge (e.g., Chiel and Beer, 1997; Clancey, 1997; Greeno, 1998). In this debate, Vera and Simon defended the compatibility of situated cognition with the ‘physical symbol system hypothesis,’ since “complex human behavior can be and has been described and simulated effectively in physical symbol systems” (Vera and Simon, 1993, p. 46).

A fundamental—still recent—counter-argument against Vera and Simon’s defense lies in the consideration that the physical symbol system hypothesis projects first-person cognitive processes onto third-person computational rules able to model them (see Clancey, 1993). By doing this it conflates the possibility of emulating, through a computer, a number of cognitive processes with a nomological necessity. In the words of Greeno and Moore (1993, p. 56): “the question should not be whether a system that uses symbolic processes is sufficient, but whether the symbolic processes that are hypothesized are necessary.”

We believe that this conflation of first- and third-person accounts is also the unwitting assumption of evolutionary psychology, where the independence between computational programs (composing a computer-like brain) and flesh-and-blood organisms represents a legitimation principle: separating computation from the body is precisely the theoretical argument that makes evolutionary psychology a domain of investigation independent from the biological realm and matters of morphology. And, interestingly, when “flesh and blood” are washed out, cross-level mechanisms (which are central in our speculation) also disappear.

Provocatively, if we endorse the view that such computational programs were sculpted by evolution, should we also assume that such programs admit artificial, out-of-the body, evolution? Can we implement such processes on a computer and simulate natural evolution through environmentally-calibrated evolutionary algorithms? We think that an embodied reconceptualization of rationality represents an alternative to the current views, which are still rooted in the cognitivist framework assumed in evolutionary psychology, where the body of the organism seems to be nothing more than hardware that is merely instrumental to the allegedly-computational processes occurring in the brain.

#### From Massive Modularity to Massive Redeployment

A fundamental tenet of evolutionary psychology is modularity, where the functional decomposition of biological systems—into functional sub-systems (e.g., organs) incrementally adapted for specific tasks—is extended to cognitive processes and endowments (Carruthers, 2006; see also Barrett and Kurzban, 2006). Innateness, along with Chomsky’s thesis on the poverty of stimulus, represents an argument in favor of the selective adaptation of cognitive faculties, as a set of evolved mechanisms that instantiate human problem solving abilities, substituting the necessity of learning everything from scratch. Such an argument endorses a specialized view of the human brain: “Natural selection ensures that the brain is composed of many different special-purpose programs and not a domain general architecture” as suggested by Tooby and Cosmides (2005, p. 17), adding that “this is a ubiquitous engineering outcome. The existence of recurrent computational problems leads to functionally specialized application software.”

A fundamental evolutionary counter-argument—central in Gould and Lewontin ‘s critique to adaptationism—is the rejection of a fixed modularity of organisms, where specialization is functionally defined. Gould and Lewontin strongly criticize the claim of adaptationism that “proceeds by breaking an organism into unitary ‘traits’ and proposing an adaptive story for each considered separately” (Gould and Lewontin, 1979, p. 581). Adaptations are generally referred to as structural (e.g., features of the human body), physiological (e.g., homeostatic mechanisms) or behavioral (e.g., inherited systems of behaviors) traits. Actually, the problem of the *unit of analysis* is crucial, since in Gould and Lewontin’s perspective we cannot make ontological distinctions but just the ones that are instrumental to evolutionary contingencies. Indeed, we have precise modularity only if we assume a congruence between structure and function, which is the precise argument criticized by Gould and Lewontin, through the notion of spandrels. Furthermore, the absence of strong empirical evidence in favor of claims about modularity suggests a need for a far more pluralistic approach (e.g., Lloyd, 1999).

Modularity (see Prinz, 2006 for a critique) enters into the rationality debate where a problematic blank-state is substituted by innateness, calling into account Darwinian evolutionary mechanisms (Samuels, 1998; Samuels et al., 1999). Moreover, this modular view is precisely the one that connotes Gigerenzer (2008)’s adaptive toolbox (see Section “Inside the Adaptive Toolbox”): “The adaptive toolbox is a Darwinian-inspired theory that conceives the mind as a modular system that is composed of heuristics, their building blocks, and evolved capacities” (p. 20), where the building blocks are precisely the cognitivist rules for symbolic manipulation—rules for search, stopping and decision (Gigerenzer and Gaissmaier, 2011).

We think that the general claim of embodied cognition—and the hypothesis of a neural reuse (discussed in Section “Neural Reuse”)—opens a quite different perspective on the evolution and nature of rationality, where neural substrates are horizontally layered instead of being vertically compartmentalized. This alternative to modularity, building on the Massive Redeployment Hypothesis, represents a significant argument for a radical updating of the current view of human rationality. Importantly, the Massive Redeployment Hypothesis does not represent a radical alternative to modularity in general terms but to such forms of modularity that, *stricto sensu*, assume domain-specificity (Anderson, 2007b).

Our view of embodied rationality, then, represents a radical alternative to the modular mind assumed by the adaptive toolbox of ecological rationality, where heuristics are domain-specific. Indeed, embodied rationality claims a horizontally layered mind—whose evolution is connoted by exaptive mechanisms of older structures—instead of an adaptive toolbox with specialized modules. Interestingly, this framework might also shed new light on the nature of automatic cognitive systems (sources of cognitive errors, in Kahneman’s view), which are plausibly more plastic and flexible than commonly assumed (see Bellini-Leite and Frankish, 2021). Its biasedness can be conceptualized as the instantiation of such evolutionary constraints from which specific reasoning processes originate.

#### Niche Construction

Evolutionary psychology emphasizes that cognitive programs (the computational rules composing the human brain) were adaptive in ancestral environments but they may not be adaptive in civilized environments (Tooby and Cosmides, 2005; Cosmides and Tooby, 2006; also see Stanovich, 2011), where the mismatch produces an *adaptive lag*. The hypothesis of the adaptive lag is plausible if we endorse a purely adaptationist view that encompasses the bottleneck of time, for the occurrence of incremental refinements. Under this hypothesis, the environment remains somehow fixed and untouched so as to identify a univocal causal direction of selective processes. As bluntly argued by Williams (1992, p. 484): “Adaptation is always asymmetrical; organisms adapt to their environment, never *vice versa.*”

Transposing this argument to the rationality debate, and using the Simon’s scissors argument, the cognitive blade adapts to the environmental blade. We think that this conception of a fixed and untouched environment in the rationality debate, might originate from Simon’s original emphasis on human problem-solving (e.g., Newell and Simon, 1972), according to which the environment is considered a mere *task environment*, representing the experimental setting used to comparatively assess human reasoning abilities (cf. Gray et al., 2006). This experimentally-operationalized environment is problematic because over decades it has forced rationality scholars to unwittingly conflate a methodological expedient into a theoretical assumption (consistently with an adaptationist perspective): organisms are problem solvers, continuously facing survival problems, out there, administered by the environment. This view is endorsed by Tooby and Cosmides (2007, p. 43, emphasis added), who stated that natural selection “favors building special assumptions, innate content, and domain-specific *problem-solving strategies* into the proprietary logic of neural devices whenever this increases their power to solve *adaptive problems.*” This conflation of a methodological expedient into a theoretical assumption is, we believe, the main cause of the marginalization of alternative evolutionary logics—in particular, niche construction—in the rationality debates.

Niche construction emphasizes the active role of the organism in manipulating the environment. In particular, the organism significantly modifies the environment thus affecting the selective processes, where such modification invites an evolutionary response of the organism (and/or other species) (Lewontin, 1983; Odling Smee et al., 2003). Niche construction represents a fundamental mechanism in the so-called ‘extended evolutionary synthesis,’ which, though retaining the fundamentals of evolutionary theory, also emphasizes the role of constructive processes in evolution and development (Laland et al., 2015)—meaning that the organism is not just the object but *the* subject of evolution. We are of course aware (and this is not the place for an extended discussion) that niche construction has been the subject of heated evolutionary debates (as in the case of exaptive processes) among proponents and critics (see Gupta et al., 2017a, and replies: Feldman et al., 2017; Gupta et al., 2017b).

The problem of the adaptive lag remains one of the points of contrast between niche construction and evolutionary psychology (Laland and Brown, 2006), a point that is also critical for the rationality debate. Indeed, evolutionary psychology remains vague on the nature of the misfit between the ancestral cognitive architecture and the civilized environment. As put by Tooby and Cosmides (2005, p. 17): “The industrial revolution—even the agricultural revolution—is too brief a period to have selected for complex new cognitive programs.” This has led many to claim that existing cognitive faculties are more and more unsuited for modern decision environments, that “the modern world tends to create situations in which the default values of evolutionarily adapted cognitive systems are not optimal” (Chater et al., 2018, p. 812). This is also an implicit assumption of the extant biases literature and it’s *de facto* claim of an “epidemic of human perceptual blindness, irrationality, and delusion” (Felin et al., 2019, p. 109). Ironically, we do not understand why we do not have a cognitive architecture adapted to civilization, but (somehow) we have cognitive structures that deliberately created such civilization. If we consider civilization a non-entropic process, a product of human deliberation (and we exclude that civilization is an accidental byproduct), why should we lack the cognitive endowments to deal with this ordered process?

Now, Cosmides and Tooby might be right that many of our cognitive endowments were adapted to the past and not to our civilized environments. However, we believe that a central matter for rationality is, precisely, what ancestral structures (and how) have been exapted to be reused to create civilized niches. From the perspective of economics (investigating advanced economic systems populated by more or less rational agents), the absence of an adaptive lag is somehow taken for granted and factual: it is of little value assuming that, say, the executive board of the European Central Bank is composed by individuals endowed with ancestral, hunter-gatherer-type cognitive architectures. Interestingly, the clash between task-relevant timescale and evolutionary timescale is central matter for neural reuse (Rathkopf, 2021). Thus we agree with Laland et al. (2007) in recommending a rejection of the adaptive-lag hypothesis “in favor of a niche-construction perspective, which focuses on how human beings respond, and are themselves responses, to self-induced environmental changes” (p. 63; also see Laland and Seed, 2021).

Cognitive arguments in favor of niche construction are abundant and central in externalist perspectives on embodied cognition (Sterelny, 2010), as the environment is *de facto* manipulated through its own artifacts so as to create the conditions which facilitate—extend and scaffold—human behavior and constrain its evolutionary paths. Cumulative technological culture continuously improves, evolves and innovates through the use of tools (e.g., Osiurak and Reynaud, 2020). The notion of ‘intoolligence’ introduces a unified framework for the cognitive study of tool use and technology, based on the general idea that making and using a tool are two independent cognitive steps (Osiurak and Heinke, 2018). The existence of cross-level exaptive mechanisms, (discussed in the Section “Cross-Level Mechanisms: Exapting the Perception-Action System”), that also imply jumps from the natural to the cultural dimension, are relevant. An unconstrained view on the unit of selection reveals the possibility of considering the interplay between nature and culture in a more flexible manner. Notice that the notion of exaptation has also been increasingly used to explain the nature of technological innovation in the economic domain, where evolutionary processes apply directly to endosomatic endowments and tools (Dew et al., 2004; Cattani, 2006; Andriani and Cattani, 2016; Felin et al., 2016; Cattani and Malerba, 2021; Cattani and Mastrogiorgio, 2021).

In short, we think that niche construction-related argument represents a fundamental opportunity for innovation for the bounded and ecological rationality literatures (see Callebaut, 2007), by stressing the relativistic and culturally-embedded criteria of normativity (e.g., Elqayam, 2011).

#### The Environment, in Place of Rationality

A central principle of evolutionary psychology is that describing the evolved computational architecture of our brains also allows us to understand cultural and social phenomena (Tooby and Cosmides, 2005). The idea is that domain-specific programs are not passive but active devices in defining our experience so as to shape cultural practices.

We think that this principle, consistent with a computational mind whose modules were adaptively selected, is problematic. Indeed, we think that cognitive architectures alone are not sufficient for understanding cultural and social phenomena. In many niches—precisely the ones of the civilized world in which rationality is paramount—the opposite consideration is also valid: social and cultural phenomena help to understand the mind. And this is not just because the environment matters as it shapes domain-specific modules by adaptive selection, but because the environment can, in a sense, operate in place of cognition. That is, a relevant perspective in the pluralism of embodied cognition approaches (see Wilson, 2002) is the externalist one, according to which the environment extends and integrates cognition: organisms do not need to gather and process information internally to the extent that they offload cognition onto the environment (Sterelny, 2010). Therefore, the external environment can be functionally equivalent to internal cognitive processes, for instance when we use calendars as external memory tools (e.g., Clark and Chalmers, 1998). And, it can also be complementary as it functionally integrates cognition, for instance when we use a pen and paper to facilitate mathematical reasoning (e.g., Menary, 2010). Moreover, the mind can be socially extended to encompass the social, institutional, and cultural dimensions (Gallagher, 2013).

Furthermore, acknowledging “others” as part of the environment helps us also to consider the role of social cognition and social learning in the development of embodied rationality. Mirror neurons are a crucial substrate in many developmental processes based on imitation (Rizzolatti et al., 2002). While many scholars see mirror neurons as a genetic adaptation for understanding action (Rizzolatti et al., 1996; Cook et al., 2014), others consider their ontogeny, specifically hypothesizing that mirror neurons are related to learning process (Giudice et al., 2009, see also Tramacere et al., 2017). Importantly, the reuse of the perception-action system is not limited to proprioception, but also involves interoception in the domain of affectivity. Higher forms of empathy, in particular mentalizing, are hypothesized to be linked to perception and motor system (specifically associated with mouth/face actions and expressions), subject to a process of exaptation during primate phylogeny (Tramacere and Ferrari, 2016). Generally speaking, social intelligence can be considered an adaptive response to the complexity of the social environment. Specifically, the different views of such complexity represent different versions of the social intelligence hypothesis (Jolly, 1966; Humphrey, 1976). In this debate, we endorse a pluralistic view on evolution where exaptive processes, also due to the unconstrained nature of the unit of selection, allow “jumps” from the genetic to the cultural domain. Hominin evolution can be seen as a response to selective environments that other hominins previously created, consistent with a niche constructionist perspective (cf. Sterelny, 2007).

With specific reference to rationality, understanding the role of social systems as external and distributed cognitive devices is crucial. The development of rationality in humans is enacted in a series of socio-cultural experiences, as the environment is significantly instantiated in interactions with others (cf. Gallagher, 2018). The enactivist approach, in particular, emphasizes the extended, intersubjective and socially situated nature of cognitive systems. From this perspective, the brain does not create an internal model of the world, but is conceptualized as a part of the larger system of brain-body-environment (for an overview see Newen et al., 2018). The existence of so-called minimal/zero intelligence agents operating in complex economic environments represents a persuasive argument against the internalist perspective (assumed in evolutionary psychology). Simple agents making elementary choices (e.g., random choices) can generate outcomes that are substantively rational (Gode and Sunder, 1993). Zero/minimal intelligence occurs precisely because some external structures take the place of an agent’s cognition. The case of embodied swarm intelligence is, in this regard, representative: elementary agents (e.g., ants) interacting at the micro-level through simple rules generate self-organized and complex societies. And such societies, in which tasks and roles are differentiated, cannot be reduced to the underlying interacting rules, being rather emergent phenomena (e.g., describing the evolved-though-rudimentary computational architecture of ants’ brains tells us little about how ants’ societies are organized).

Modernity is populated by devices, and whole cultural systems, that work in place of individuals’ cognition. Indeed, zero/minimal intelligence could also be conceptualized in terms of an institutional perspective (Hodgson, 2004; Felin, 2015; Petracca and Gallagher, 2020), where economic institutions work in place of internal cognitive processes, doing most of the cognitive job by scaffolding agents’ decisions and actions (Clark, 1997). Externalizing the cognitive burden is not just a matter of sharing mental models among the economic agents as suggested by Denzau and North (1994), but calls into account the whole existence of so-called cognitive institutions (Gallagher and Crisafi, 2009; Petracca and Gallagher, 2020). Interestingly, markets—composed by a collectivity of (more or less) rational agents—are precisely such cultural devices that offer an ostensive counter-argument to the evolutionary psychologists’ claims about a pure internalist perspective. Markets can be considered as socially extended institutions able to solve collective allocation problems unsolvable by agents with an internalized and disembodied rationality. Markets are social institutions that emerge from intersubjective “embodied” interactions, producing a “cognitive economy” as they reduce individual cognitive effort in making decisions and enable specific types of economic reasoning processes (for a discussion see Gallagher et al., 2019).

## Conclusion

According to the philosopher Wittgenstein (1953) the rules of language are analogous to the rules of games, where words are ambiguous and have a meaning only within the specific linguistic game being played: words do not point to ontologically fixed entities but they are merely instrumental to contingent interaction. In this contribution, ‘thumbs’ are such an ambiguous word, contingent upon the narrative. Thumbs are exemplifications of what we mean by heuristics (as rules of “thumbs”), they are effects of exaptive processes (as in the case of a small bone of the wrist, exapted to became the panda’s opposable thumb), and they are enabling constraints (as in the case of finger gnosis that enables numerical processing).

Our use of ‘thumb’ therefore is not just a rhetorically expedient device for scientific communication but uncovers an evolutionary matter central to our arguments. Exaptive mechanisms require us to disentangle structure and function to the extent that their interplay cannot be pre-stated, but rather represents precisely a point which evolutionary forces apply to. As in the case of the feathers of birds (first evolved for thermal regulation and then co-opted for flight), functional ambiguity is probably a property of evolution that is able to (re)attribute contingent meanings to biological structures. And this process relativizes the possibility of framing evolutionary units in once-for-all, ontologically-defined categories (Kauffman, 2000, 2008; Longo et al., 2012; Roli and Kauffman, 2020). And at a high level of abstraction, the whole process of evolution toward rationality might be seen as adjacent possibility, emergent—contingently, through reuse—from the embodied endowments of the organism co-evolved with a continually changing environment.

In this contribution, we propose an embodied reconceptualization of rationality—*embodied rationality*—based on the reuse of the perception-action system. We argue that neural processes involved in the control of the sensory-motor system, salient in ancestral environments, can be co-opted to create (by tinkering) high-level cognitive faculties, employed in civilized niches. The idea that ‘rationality is an exaptation’ is actually an exemplification, as rationality, in our view, is not a unitary system but is made of a stratified mix of many exaptations of the sensory-motor system on which higher cognitive processes are grounded (the exaptation of finger gnosis registers, discussed in Section “The Case of Fingers in Numerical Cognition” is only one of them. Notice that we use the expression ‘sensory-motor system’ in the singular form as an exemplification, but we actually assume that there is a multitude of exapted systems). To revisit Gould and Lewontin’s architectural, “spandrels” metaphor, the cognitive architecture of embodied rationality does not resemble a futuristic and optimized building, as much of evolutionary psychology suggests. Rather, cognition resembles a harmonious, pleasant and effective jumble of stratified architectural structures (similar to Tuscan farmhouses), the products of tinkering. In this stratified architecture, the many spandrels and byproducts matter a great deal in defining the overall result and the adjacent evolutionary possibilities.

But, considering exaptive mechanism a *tout court* alternative to adaptive logics is wrong. Exaptive processes do not rule out adaptive mechanisms—they do not imply a paradigm-shift, *stricto sensu*, on evolution—rather they extend the richness of evolutionary possibilities. Thus, embodied rationality does not represent an alternative to bounded and ecological rationality but precisely an integration, aiming to challenge and update the underlying adaptationist and cognitivist assumptions. However, the innovativeness of our proposal does not rely on a *tout-court* redefinition of rationality, and *a fortiori*, does not imply to identify a novel and different type of heuristics. Nothing changes in the performative dimension of rationality.

The relevant innovation that *embodied rationality* brings to the debate is related to the novel role of the human body, and the attendant notions of bodily, cognitive and material reuse. While bounded and ecological rationality (flourished in the cognitivist paradigm) prescind from the biological endowments, embodied rationality emphasizes the constitutive dependence of heuristics on the human body and in particular on the sensory-motor system. Actually, bounded and ecological rationality do not rule out embodiment in absolute terms: on the one side a number of heuristics of the adaptive toolbox (such as the gaze heuristic, e.g., Raab and Gigerenzer, 2005) require the use of the sensory-motor system, on the other side, gut feelings are determinant for heuristic choice (e.g., Gigerenzer, 2007). Despite such embodied arguments, heuristics in bounded and ecological rationality remain disembodied in the sense that human body plays the role of a neutral hardware on which the cognitive software runs.

That is to say, heuristics are seen as algorithmic rules for information processing, which could hypothetically run on various types of bodily hardware (cf. Simon, 1990). Embodied rationality, on the contrary, claims the non-neutrality of biological endowment for the specification of the cognitive processes, and this argument represents a distinctive mark of embodied rationality, which cannot be found in ecological and bounded rationality.

Hence, embodied rationality invites us to abandon a third-person rationality (where cognitive processes can be expressed as objectified, algorithmic rules for information processing) and calls into account the biological realm. That is, high-level cognitive processes can be understood precisely as they are grounded on the sensory-motor system, and not prescinding from it, where such grounding can be considered the pivot of Simon’s scissor. Such grounding allows us to account for the origins of heuristics. While bounded and ecological rationality have offered us different types of heuristics, they are not able to explain how heuristics came into being. Embodied rationality can be useful to ascribe the origins of heuristics to specific evolutionary constraints that specify the adjacent possible for cognition—which cognitive processes are “affordable” by neural reuse. Investigating the ontogenetic and phylogenetic dimensions, along with task-relevant or evolutionary timescales of neural reuse, represent a future domain of investigation for embodied rationality.

## Data Availability Statement

The original contributions presented in the study are included in the article/supplementary material, further inquiries can be directed to the corresponding author.

## Author Contributions

AM: conceptualization, supervision, writing – original draft, and writing – reviewing and editing. TF: conceptualization and writing – reviewing and editing. SK and MM: conceptualization and reviewing. All authors contributed to the article and approved the submitted version.

## Conflict of Interest

The authors declare that the research was conducted in the absence of any commercial or financial relationships that could be construed as a potential conflict of interest.

## Publisher’s Note

All claims expressed in this article are solely those of the authors and do not necessarily represent those of their affiliated organizations, or those of the publisher, the editors and the reviewers. Any product that may be evaluated in this article, or claim that may be made by its manufacturer, is not guaranteed or endorsed by the publisher.

## References

[B1] Amitani Y. (2015). The natural frequency hypothesis and evolutionary arguments. *Mind Soc.* 14 1–19. 10.1007/s11299-014-0155-7

[B2] Anderson M. L. (2006). Evidence for massive redeployment of brain areas in cognitive function. *Proc. Cogn. Sci. Soc.* 28 24–29.

[B3] Anderson M. L. (2007a). Massive redeployment, exaptation, and the functional integration of cognitive operations. *Synthese* 159 329–345. 10.1007/s11229-007-9233-2

[B4] Anderson M. L. (2007b). The massive redeployment hypothesis and the functional topography of the brain. *Philos. Psychol.* 20 143–174. 10.1080/09515080701197163

[B5] Anderson M. L. (2008). “On the grounds of (X)-grounded cognition,” in *Handbook of Cognitive Science: An Embodied Approach*, eds Calvo P. Gomila T. (Amsterdam: Elsevier), 423–435. 10.1016/B978-0-08-046616-3.00021-9

[B6] Anderson M. L. (2010). Neural reuse: a fundamental organizational principle of the brain. *Behav. Brain Sci.* 33 245–266. 10.1017/S0140525X10000853 20964882

[B7] Anderson M. L. (2014). *After Phrenology: Neural Reuse and the Interactive Brain.* Cambridge, MA: MIT Press. 10.7551/mitpress/10111.001.0001 26077688

[B8] Anderson M. L. (2016). Précis of after phrenology: neural reuse and the interactive brain. *Behav. Brain Sci.* 39:e120. 10.1017/S0140525X15000631 26077688

[B9] Anderson M. L. Kinnison J. Pessoa L. (2013). Describing functional diversity of brain regions and brain networks. *Neuroimage* 73 50–58. 10.1016/j.neuroimage.2013.01.071 23396162 PMC3756684

[B10] Andrews P. W. Gangestad S. W. Matthews D. (2002). Adaptationism-how to carry out an exaptationist program. *Behav. Brain Sci.* 25 489–504. 10.1017/S0140525X02000092 12879701

[B11] Andriani P. Cattani G. (2016). Exaptation as source of creativity, innovation, and diversity: introduction to the special section. *Indust. Corp. Change* 25 115–131. 10.1093/icc/dtv053 30476010

[B12] Barack D. L. (2017). Cognitive recycling. *Br. J. Philos. Sci.* 70 239–268. 10.1093/bjps/axx024 32691291

[B13] Barrett H. C. Kurzban R. (2006). Modularity in cognition: framing the debate. *Psychol. Rev.* 113 628–647. 10.1037/0033-295X.113.3.628 16802884

[B14] Bellini-Leite S. C. Frankish K. (2021). “Bounded rationality and dual systems,” in *Handbook of Bounded Rationality*, ed. Viale R. (London: Routledge), 207–216. 10.4324/9781315658353-13

[B15] Bueti D. Walsh V. (2009). The parietal cortex and the representation of time, space, number and other magnitudes. *Philos. Trans. R. Soc. B Biol. Sci.* 364 1831–1840. 10.1098/rstb.2009.0028 19487186 PMC2685826

[B16] Buss D. M. Haselton M. G. Shackelford T. K. Bleske A. L. Wakefield J. C. (1998). Adaptations, exaptations, and spandrels. *Am. Psychol.* 53:533. 10.1037/0003-066X.53.5.533 9612136

[B17] Callebaut W. (2007). Simon’s silent revolution. *Biol. Theory* 2 76–86. 10.1162/biot.2007.2.1.76

[B18] Calvo P. Gomila T. (eds) (2008). *Handbook of Cognitive Science: An Embodied Approach.* Amsterdam: Elsevier.

[B19] Campbell D. T. (1960). Blind variation and selective retention in creative thought as in other knowledge processes. *Psychol. Rev.* 67 380–400. 10.1037/h0040373 13690223

[B20] Carruthers P. (2006). *The Architecture of the Mind.* Oxford: Clarendon Press. 10.1093/acprof:oso/9780199207077.001.0001

[B21] Cattani G. (2006). Technological pre-adaptation, speciation, and emergence of new technologies: how corning invented and developed fiber optics. *Ind. Corp. Change* 15, 285–318. 10.1093/icc/dtj016 30476010

[B22] Cattani G. Malerba F. (2021). Evolutionary approaches to innovation, the firm, and the dynamics of industries. *Strategy Sci.* 6, 265–289. 10.1287/stsc.2021.0141 19642375

[B23] Cattani G. Mastrogiorgio M. (eds) (2021). *New Developments in Evolutionary Innovation: Novelty Creation in a Serendipitous Economy.* Oxford: Oxford University Press. 10.1093/oso/9780198837091.001.0001

[B24] Cermak S. A. Larkin D. (2001). *Developmental Coordination Disorder.* Albany, NY: Delmar.

[B25] Chapman P. D. Bradley S. P. Haught E. J. Riggs K. E. Haffar M. M. Daly K. C. (2017). Co-option of a motor-to-sensory histaminergic circuit correlates with insect flight biomechanics. *Proc. R. Soc. B Biol. Sci.* 284:20170339. 10.1098/rspb.2017.0339 28747471 PMC5543211

[B26] Chater N. Felin T. Funder D. C. Gigerenzer G. Koenderink J. J. Krueger J. I. (2018). Mind, rationality, and cognition: an interdisciplinary debate. *Psychonom. Bull. Rev.* 25 793–826. 10.3758/s13423-017-1333-5 28744767 PMC5902517

[B27] Chiel H. Beer R. (1997). The brain has a body: adaptive behavior emerges from interactions of nervous system, body, and environment. *Trends Neurosci.* 20 553–557. 10.1016/S0166-2236(97)01149-1 9416664

[B28] Chiou R. Y. C. Wu D. H. Tzeng O. J. L. Hung D. L. Chang E. C. (2012). Relative size of numerical magnitude induces a size-contrast effect on the grip scaling of reach-to-grasp movements. *Cortex* 48 1043–1051. 10.1016/j.cortex.2011.08.001 21889134

[B29] Clancey W. (1997). *Situated Cognition: On Human Knowledge and Computer Representations.* Cambridge, MA: Cambridge University Press.

[B30] Clancey W. J. (1993). Situated Action: a neuropsychological interpretation (Response to Vera and Simon). *Cogn. Sci.* 17 117–133. 10.1207/s15516709cog1701_7

[B31] Clark A. (1997). “Economic reason: the interplay of individual learning and external structure,” in *The Frontiers of the New Institutional Economics*, eds Drobak J. Nye J. (San Diego, CA: Academic Press), 269–290.

[B32] Clark A. (2008). Pressing the flesh: a tension in the study of the embodied, embedded mind? *Philos. Phenomenol. Res.* 76 36–59. 10.1111/j.1933-1592.2007.00114.x

[B33] Clark A. Chalmers D. (1998). The extended mind. *Analysis* 58 7–19. 10.1093/analys/58.1.7

[B34] Cohen Kadosh R. Walsh V. (2009). Numerical representation in the parietal lobes: Abstract or not abstract? *Behav. Brain Sci.* 32 313–373. 10.1017/S0140525X09990938 19712504

[B35] Cook R. Bird G. Catmur C. Press C. Heyes C. (2014). Mirror neurons: from origin to function. *Behav. Brain Sci.* 37 177–192. 10.1017/S0140525X13000903 24775147

[B36] Cosmides L. Tooby J. (2006). *Evolutionary Psychology, Moral Heuristics, and the Law.* Oxford: Dahlem University Press. 10.1002/0470018860.s00529

[B37] Cosmides L. Tooby J. (2013). Evolutionary psychology: new perspectives on cognition and motivation. *Ann. Rev. Psychol.* 64 201–229. 10.1146/annurev.psych.121208.131628 23282055

[B38] Darwin C. (1872). *The Origin of Species.* London: John Murray. 10.5962/bhl.title.28875

[B39] Dawkins R. (1983). “Universal darwinism,” in *Evolution from Molecules to Man*, ed. Bendall D. S. (Cambridge: Cambridge University Press).

[B40] Dehaene S. Cohen L. (2007). Cultural recycling of cortical maps. *Neuron* 56 384–398. 10.1016/j.neuron.2007.10.004 17964253

[B41] Dehaene S. Dehaene-Lambertz G. Cohen L. (1998). Abstract representations of numbers in the animal and human brain. *Trends Neurosci.* 21 355–361. 10.1016/S0166-2236(98)01263-6 9720604

[B42] Dennett D. (1997). Darwinian fundamentalism: an exchange. *N. Y. Rev. Books* 44 13.

[B43] Denzau A. T. North D. C. (1994). Shared mental models: Ideologies and institutions. *Kyklos* 47 3–31. 10.1111/j.1467-6435.1994.tb02246.x

[B44] Dew N. Sarasvathy S. D. Venkataraman S. (2004). The economic implications of exaptation. *J. Evol. Econ.* 14 69–84. 10.1007/s00191-003-0180-x

[B45] Dhami M. K. Hertwig R. Hoffrage U. (2004). The role of representative design in an ecological approach to cognition. *Psychol. Bull.* 130 959–988. 10.1037/0033-2909.130.6.959 15535744

[B46] Eldredge N. Gould S. J. (1972). “Punctuated equilibria: an alternative to phyletic gradualism,” in *Models in Paleobiology*, ed. Schopf T. J. M. (San Francisco, CA: Freeman, Cooper and Co), 82–115. 10.5531/sd.paleo.7

[B47] Elqayam S. (2011). “Grounded rationality: a relativist framework for normative rationality,” in *The Science of Reason: A Festschrift in Honour of Jonathan St.B.T. Evans*, eds Manktelow K. I. Over D. E. Elqayam S. (Hove: Psychology Press).

[B48] Favela L. H. (2021). “Fundamental theories in neuroscience: why neural darwinism encompasses neural reuse,” in *Neural Mechanisms. Studies in Brain and Mind*, Vol. 17 eds Calzavarini F. Viola M. (Cham: Springer), 143–162. 10.1007/978-3-030-54092-0_7

[B49] Feldman M. W. Odling-Smee J. Laland K. N. (2017). Why Gupta et al’s critique of niche construction theory is off target. *J. Genet.* 96 505–508. 10.1007/s12041-017-0797-4 28761013

[B50] Felin T. (2015). A forum on minds and institutions. *J. Instit. Econ.* 11 523–534. 10.1017/S1744137415000144

[B51] Felin T. Felin M. Krueger J. I. Koenderink J. (2019). On surprise-hacking. *Perception* 48 109–114. 10.1177/0301006618822217 30606069

[B52] Felin T. Kauffman S. Mastrogiorgio A. Mastrogiorgio M. (2016). Factor markets, actors, and affordances. *Indus. Corp. Chang.* 25 133–147. 10.1093/icc/dtv049 30476010

[B53] Frederick S. (2005). Cognitive reflection and decision making. *J. Econ. Perspect.* 19 25–42. 10.1257/089533005775196732

[B54] Gallagher S. (2013). The socially extended mind. *Cogn. Syst. Res.* 25 4–12. 10.1016/j.cogsys.2013.03.008

[B55] Gallagher S. (2018). “Embodied rationality,” in *The Mystery of Rationality*, eds Bronner G. Di Iorio F. (Cham: Springer), 83–94. 10.1007/978-3-319-94028-1_7

[B56] Gallagher S. Crisafi A. (2009). Mental institutions. *Topoi* 28 45–51. 10.1007/s11245-008-9045-0

[B57] Gallagher S. Mastrogiorgio A. Petracca E. (2019). Economic reasoning and interaction in socially extended market institutions. *Front. Psychol.* 10:1856. 10.3389/fpsyg.2019.01856 31551841 PMC6736591

[B58] Gallese V. (2008). Mirror neurons and the social nature of language: the neural exploitation hypothesis. *Soc. Neurosci.* 3 317–333. 10.1080/17470910701563608 18979384

[B59] Gallese V. Lakoff G. (2005). The brain’s concepts: the role of the sensorimotor system in reason and language. *Cogn. Neuropsychol.* 22 455–479. 10.1080/02643290442000310 21038261

[B60] Gallese V. Mastrogiorgio A. Petracca E. Viale R. (2021). “Embodied bounded rationality,” in *Handbook of Bounded Rationality*, ed. Viale R. (London: Routledge). 10.4324/9781315658353-26

[B61] Gigerenzer G. (1991). “How to make cognitive illusions disappear: beyond “heuristics and biases,” in *European Review of Social Psychology*, Vol. 2 eds Stroebe W. Hewstone M. (Chichester: Wiley), 83–115. 10.1080/14792779143000033

[B62] Gigerenzer G. (1996). On narrow norms and vague heuristics: a reply to Kahneman and Tversky. *Psychol. Rev.* 103 592–596. 10.1037/0033-295X.103.3.592

[B63] Gigerenzer G. (2007). *Gut Feelings: The Intelligence of the Unconscious.* London: Penguin.

[B64] Gigerenzer G. (2008). Why heuristics work. *Perspect. Psychol. Sci.* 3 20–29. 10.1111/j.1745-6916.2008.00058.x 26158666

[B65] Gigerenzer G. (2019). How to explain behavior? *Top. Cogn. Sci.* 12 1363–1381. 10.1111/tops.12480 31692281

[B66] Gigerenzer G. Brighton H. (2009). Homo heuristicus: why biased minds make better inferences. *Top. Cogn. Sci.* 1, 107–143. 10.1111/j.1756-8765.2008.01006.x 25164802

[B67] Gigerenzer G. Gaissmaier W. (2011). Heuristic decision making. *Ann. Rev. Psychol.* 62 451–482. 10.1146/annurev-psych-120709-145346 21126183

[B68] Gigerenzer G. Goldstein D. G. (1996). Reasoning the fast and frugal way: models of bounded rationality. *Psychol. Rev.* 103 650–669. 10.1037/0033-295X.103.4.650 8888650

[B69] Gigerenzer G. Gray W. D. (2017). A simple heuristic successfully used by humans, animals, and machines: the story of the RAF and Luftwaffe, hawks and ducks, dogs and frisbees, baseball outfielders and sidewinder missiles-oh my! *Top. Cogn. Sci.* 9 260–263. 10.1111/tops.12269 28382679

[B70] Gigerenzer G. Hoffrage U. (1995). How to improve Bayesian reasoning without instruction: frequency formats. *Psychol. Rev.* 102 684–704. 10.1037/0033-295X.102.4.684

[B71] Gigerenzer G. Murray D. J. (1987). *Cognition as Intuitive Statistics.* Hillsdale, NJ: Erlbaum.

[B72] Gigerenzer G. Murray D. J. (2015). *Cognition as Intuitive Statistics.* Hove: Psychology Press. 10.4324/9781315668796

[B73] Gigerenzer G. Selten R. (eds) (2002). *Bounded Rationality: The Adaptive Toolbox.* Cambridge, MA: MIT press. 10.7551/mitpress/1654.001.0001

[B74] Giudice M. D. Manera V. Keysers C. (2009). Programmed to learn? The ontogeny of mirror neurons. *Dev. Sci.* 12 350–363. 10.1111/j.1467-7687.2008.00783.x 19143807

[B75] Gode D. K. Sunder S. (1993). Allocative efficiency of markets with zero-intelligence traders: Market as a partial substitute for individual rationality. *J. Polit. Econ.* 101 119–137. 10.1086/261868

[B76] Gould S. J. (1982). Darwinism and the expansion of evolutionary theory. *Science* 216 380–387. 10.1126/science.7041256 7041256

[B77] Gould S. J. (1991). Exaptation: a crucial tool for an evolutionary psychology. *J. Soc. Iss.* 47 43–65. 10.1111/j.1540-4560.1991.tb01822.x

[B78] Gould S. J. (1997). Darwinian fundamentalism. *N. Y. Rev. Books* 44 34–37.

[B79] Gould S. J. (2002). *The Structure of Evolutionary Theory.* Cambridge, MA: Belknap-Harvard. 10.2307/j.ctvjsf433

[B80] Gould S. J. Eldredge N. (1977). Punctuated equilibria: the tempo and mode of evolution reconsidered. *Paleobiology* 3, 115–151.

[B81] Gould S. J. Lewontin R. C. (1979). The spandrels of San Marco and the Panglossian paradigm: a critique of the adaptationist programme. *Proc. R. Soc. Lon. Ser. B. Biol. Sci.* 205 581–598. 10.1098/rspb.1979.0086 42062

[B82] Gould S. J. Vrba E. (1982). Exaptation - a missing term in the science of form. *Paleobiology* 8 4–15. 10.1017/S0094837300004310

[B83] Gray W. D. Neth H. Schoelles M. J. (2006). “The functional task environment,” in *Attention: From Theory to Practice*, eds Kramer A. F. Wiegmann D. A. Kirlik A. (Oxford: Oxford University Press), 100–118. 10.1093/acprof:oso/9780195305722.003.0008

[B84] Greeno J. (1998). The situativity of knowing, learning, and research. *Am. Psychol.* 53 5–26. 10.1037/0003-066X.53.1.5

[B85] Greeno J. C. Moore J. L. (1993). Situativity and symbols: response to vera and simon. *Cogn. Sci.* 17 49–59. 10.1207/s15516709cog1701_3

[B86] Gupta M. Prasad N. G. Dey S. Joshi A. Vidya T. N. C. (2017a). Niche construction in evolutionary theory: the construction of an academic niche? *J. Genet.* 96 491–504. 10.1007/s12041-017-0787-6 28761012

[B87] Gupta M. Prasad N. G. Dey S. Joshi A. Vidya T. N. C. (2017b). Feldman do protest too much, we think. *J. Genet.* 96 509–511. 10.1007/s12041-017-0796-5 28761014

[B88] Hamlin R. P. (2017). The gaze heuristic:” biography of an adaptively rational decision process. *Top. Cogn. Sci.* 9 264–288. 10.1111/tops.12253 28220988

[B89] Hertwig R. Gigerenzer G. (1999). The ‘conjunction fallacy’ revisited: how intelligent inferences look like reasoning errors. *J. Behav. Decis. Making* 12, 275–305.

[B90] Hertwig R. Pleskac T. J. (2010). Decisions from experience: why small samples? *Cognition* 115 225–237. 10.1016/j.cognition.2009.12.009 20092816

[B91] Hodgson G. M. (2004). *The Evolution of Institutional Economics.* London: Routledge. 10.4324/9780203300350

[B92] Hodgson G. M. (2005). Generalizing Darwinism to social evolution: some early attempts”. *J. Econ. Iss.* 39 899–914. 10.1080/00213624.2005.11506859

[B93] Höfer S. Raisch J. Toussaint M. Brock O. (2018). No free lunch in ball catching: a comparison of Cartesian and angular representations for control. *PLoS One* 13:e0197803. 10.1371/journal.pone.0197803 29902180 PMC6002113

[B94] Humphrey N. (1976). “The social function of intellect,” in *Growing Points in Ethology*, eds Bateson P. P. G. Hinde R. A. (Cambridge: Cambridge University Press), 303–317.

[B95] Hutchinson J. M. Gigerenzer G. (2005). Simple heuristics and rules of thumb: where psychologists and behavioural biologists might meet. *Behav. Process.* 69 97–124. 10.1016/j.beproc.2005.02.019 15845293

[B96] Jacob F. (1977). Evolution and tinkering. *Science* 196 1161–1166. 10.1126/science.860134 860134

[B97] Jolly A. (1966). Lemur social behavior and primate intelligence. *Science* 153 501–506. 10.1126/science.153.3735.501 5938775

[B98] Kaaronen R. O. (2020). Mycological rationality: heuristics, perception and decision-making in mushroom foraging. *Judg. Dec. Mak.* 15 630–647. 10.31234/osf.io/7g8er

[B99] Kahneman D. (2011). *Thinking, Fast and Slow.* Basingstoke: Macmillan.

[B100] Kahneman D. Slovic P. Tversky A. (1982). *Judgment Under Uncertainty: Heuristics and Biases.* Cambridge: Cambridge University Press. 10.1017/CBO9780511809477 17835457

[B101] Kahneman D. Tversky A. (1996). On the reality of cognitive illusions: a reply to Gigerenzer’s critique. *Psychol. Rev.* 103 582–591. 10.1037/0033-295X.103.3.582 8759048

[B102] Kalant H. Pinker S. Kalow W. (1997). Evolutionary psychology: an exchange. *Exchange* 44 55–58.

[B103] Karelaia N. Hogarth R. M. (2008). Determinants of linear judgment: a meta-analysis of lens model studies. *Psychol. Bull.* 134 404–426. 10.1037/0033-2909.134.3.404 18444703

[B104] Kauffman S. A. (2000). *Investigations.* Oxford: Oxford University Press.

[B105] Kauffman S. A. (2008). *Reinventing the Sacred: A New View of Science, Reason, and Religion.* New York, NY: Basic Books.

[B106] Kiverstein J. Clark A. (2009). Introduction: mind embodied, embedded, enacted: one church or many? *Topoi* 28 1–7. 10.1007/s11245-008-9041-4

[B107] Lakoff G. Núñez R. (2000). *Where Mathematics Comes From: How the Embodied Mind Brings Mathematics into Being.* New York, NY: Basic Books.

[B108] Laland K. Seed A. (2021). Understanding human cognitive uniqueness. *Annu. Rev. Psychol.* 72 689–716. 10.1146/annurev-psych-062220-051256 33400565

[B109] Laland K. N. Brown G. R. (2006). Niche construction, human behavior, and the adaptive-lag hypothesis. *Evol. Anthropol.* 15 95–104. 10.1002/evan.20093

[B110] Laland K. N. Kendal J. R. Brown G. R. (2007). The niche construction perspective: Implications for evolution and human behaviour. *J. Evol. Psychol.* 5 51–66. 10.1556/JEP.2007.1003 24564590

[B111] Laland K. N. Uller T. Feldman M. W. Sterelny K. Müller G. B. Moczek A. (2015). The extended evolutionary synthesis: its structure, assumptions and predictions. *Proc. R. Soc. B Biol. Sci.* 282:20151019. 10.1098/rspb.2015.1019 26246559 PMC4632619

[B112] Lewontin R. C. (1970). The units of selection. *Ann. Rev. Ecol. Syst.* 1 1–18. 10.1146/annurev.es.01.110170.000245

[B113] Lewontin R. C. (1983). “Gene, organism and environment,” in *Evolution from Molecules to Men*, ed. Bendall D. S. (Cambridge: Cambridge University Press).

[B114] Lloyd E. A. (1999). Evolutionary psychology: the burdens of proof. *Biol. Philos.* 14 211–233. 10.1023/A:1006638501739

[B115] Lloyd E. A. Gould S. J. (2017). Exaptation revisited: changes imposed by evolutionary psychologists and behavioral biologists. *Biol. Theory* 12 50–65. 10.1007/s13752-016-0258-y

[B116] Longo G. Montévil M. Kauffman S. (2012). “No entailing laws, but enablement in the evolution of the biosphere,” in *Proceedings of the 14th Annual Conference Companion on Genetic and Evolutionary Computation* (New York, NY), 1379–1392. 10.1145/2330784.2330946

[B117] Luan S. Schooler L. J. Gigerenzer G. (2014). From perception to preference and on to inference: an approach–avoidance analysis of thresholds. *Psychol. Rev.* 121 501–525. 10.1037/a0037025 25090429

[B118] Marcus G. F. (2008). *Kluge: The Haphazard Construction of the Human Mind.* Boston, MA: Houghton Mifflin.

[B119] Marewski J. N. Gigerenzer G. (2012). Heuristic decision making in medicine. *Dial. Clin. Neurosci.* 14:77. 10.31887/DCNS.2012.14.1/jmarewski 22577307 PMC3341653

[B120] Mastrogiorgio A. (2011). “The embodied dimension of rationality: a hypothesis,” in *Proceedings of the IAREP/SABE/ICABEEP Conference 2011* (England: University of Exeter),

[B121] Mastrogiorgio A. (2015). Commentary: cognitive reflection versus calculation in decision making. *Front. Psychol.* 6:936. 10.3389/fpsyg.2015.00532 26191032 PMC4490207

[B122] Mastrogiorgio A. Petracca E. (2012). “Setting the ground for a theory of embodied rationality,” in *Proceedings of the IAREP*, Vol. 2012 eds Gasiorowska A. Zaleskiewicz T. (Wrocklaw), 201.

[B123] Mastrogiorgio A. Petracca E. (2014). Numerals as triggers of system 1 and system 2 in the ‘bat and ball’ problem. *Mind Soc.* 13 135–148. 10.1007/s11299-014-0138-8

[B124] Mastrogiorgio A. Petracca E. (2015). Razionalità incarnata. *Sist. Intell.* 27 481–504.

[B125] Mastrogiorgio A. Petracca E. (2016). “Embodying rationality,” in *Model-Based Reasoning in Science and Technology*, eds Magnani L. Casadio C. (Cham: Springer), 219–237. 10.1007/978-3-319-38983-7_12

[B126] Menary R. (2010). “Cognitive integration and the extended mind,” in *The Extended Mind*, ed. Menary R. (Cambridge, MA: The MIT Press). 10.7551/mitpress/9780262014038.001.0001

[B127] Navarrete G. Santamaría C. (2011). Ecological rationality and evolution: the mind really works that way? *Front. Psychol.* 2:251. 10.3389/fpsyg.2011.00251 21994499 PMC3183440

[B128] Newell A. Simon H. A. (1972). *Human Problem Solving.* Englewood Cliffs, NJ: Prentice Hall.

[B129] Newell A. Simon H. A. (1976). Computer science as empirical inquiry: symbols and search. *Commun. ACM* 19 113–126. 10.1145/360018.360022

[B130] Newen A. De Bruin L. Gallagher S. (2018). *The Oxford Handbook of 4E Cognition.* Oxford: Oxford University Press. 10.1093/oxfordhb/9780198735410.001.0001

[B131] Odling Smee J. Laland K. Feldman M. (2003). *Niche Construction: The Neglected Process in Evolution.* Princeton, NJ: Princeton University Press, 488.

[B132] Osiurak F. Heinke D. (2018). Looking for intoolligence: a unified framework for the cognitive study of human tool use and technology. *Am. Psychol.* 73:169. 10.1037/amp0000162 29355355

[B133] Osiurak F. Reynaud E. (2020). The elephant in the room: what matters cognitively in cumulative technological culture. *Behav. Brain Sci.* 43:e156. 10.1017/S0140525X19003236 31739823

[B134] Parkinson C. Wheatley T. (2015). The repurposed social brain. *Trends Cogn. Sci.* 19 133–141. 10.1016/j.tics.2015.01.003 25732617

[B135] Penner-Wilger M. Anderson M. L. (2008). “An alternative view of the relation between finger gnosis and math ability: Redeployment of finger representations for the representation of number,” in *Proceedings of the 30th Annual Conference of the Cognitive Science Society*, eds Sloutsky V. Love B. McRae K. (Austin, TX: Cognitive Science Society).

[B136] Petracca E. (2021). Embodying bounded rationality: from embodied bounded rationality to embodied rationality. *Front. Psychol.* 12:710607. 10.3389/fpsyg.2021.710607 34566788 PMC8459109

[B137] Petracca E. Gallagher S. (2020). Economic cognitive institutions. *J. Inst. Econ.* 16 747–765. 10.1017/S1744137420000144

[B138] Pievani T. Serrelli E. (2011). Exaptation in human evolution: how to test adaptive vs exaptive evolutionary hypotheses. *J. Anthropol. Sci.* 89 9–23. 21757789 10.4436/jass.89015

[B139] Pigliucci M. Müller G. B. (eds) (2010). *Evolution - the Extended Synthesis.* Cambridge, MA: The MIT Press. 10.7551/mitpress/9780262513678.001.0001

[B140] Pinker S. (2002). *The Blank Slate.* New York, NY: Viking Press.

[B141] Pittendrigh C. S. (1958). “Adaptation, natural selection, and behavior,” in *Behavior and Evolution*, eds Roe A. Simpson G. G. (New Haven, CT: Yale University Press), 390–416.

[B142] Poldrack R. A. (2006). Can cognitive processes be inferred from neuroimaging data? *Trends Cogn. Sci.* 10 59–63. 10.1016/j.tics.2005.12.004 16406760

[B143] Prinz J. J. (2006). Is the mind really modular. *Contemp. Debates Cogn. Sci.* 14 22–36.

[B144] Raab M. Gigerenzer G. (2005). “Intelligence as smart heuristics,” in *Cognition and Intelligence*, eds Sternberg R. J. Pretz J. E. (Cambridge: Cambridge University Press), 188–207.

[B145] Ranzini M. Lugli L. Anelli F. Carbone R. Nicoletti R. Borghi A. M. (2011). Graspable objects shape number processing. *Front. Hum. Neurosci.* 5:147. 10.3389/fnhum.2011.00147 22164141 PMC3230823

[B146] Rathkopf C. (2021). “Neural reuse and the nature of evolutionary constraints,” in *Neural Mechanisms. Studies in Brain and Mind*, Vol. 17 eds Calzavarini F. Viola M. (Cham: Springer), 191–208. 10.1007/978-3-030-54092-0_9

[B147] Rizzolatti G. Fadiga L. Fogassi L. Gallese V. (2002). “From mirror neurons to imitation, facts, and speculations,” in *The Imitative mind: Development, Evolution, and Brain Bases*, eds Meltzoff A. N. Prinz W. (Cambridge: Cambridge University Press), 247–266. 10.1017/CBO9780511489969.015

[B148] Rizzolatti G. Fadiga L. Gallese V. Fogassi L. (1996). Premotor cortex and the recognition of motor actions. *Cogn. Brain Res.* 3 131–141. 10.1016/0926-6410(95)00038-0 8713554

[B149] Roli A. Kauffman S. A. (2020). Emergence of organisms. *Entropy* 22:1163. 10.3390/e22101163 33286932 PMC7597334

[B150] Samuels R. (1998). Evolutionary psychology and the massive modularity hypothesis. *Br. J. Philos. Sci.* 49 575–602. 10.1093/bjps/49.4.575 32691291

[B151] Samuels R. Stich S. Bishop M. (2004). “Ending the rationality wars: how to make disputes about human rationality disappear,” in *Common Sense, Reasoning, and Rationality*, ed. Renee E. (New York, NY: Oxford University Press), 236–268. 10.1093/0195147669.003.0011

[B152] Samuels R. Stich S. Tremoulet P. D. (1999). “Rethinking rationality: from bleak implications to darwinian modules,” in *What is Cognitive Science?*, eds Lepore E. Pylyshyn Z. (Oxford: Blackwell), 74–120. 10.1007/978-94-017-1070-1_3

[B153] Sanabria F. Killeen P. R. (2005). All thumbs? *Behav. process.* 69 143–145. 10.1016/j.beproc.2005.02.015 15845300

[B154] Sato M. Cattaneo L. Rizzolatti G. Gallese V. (2007). Numbers within our hands: Modulation of corticospinal excitability of hand muscles during numerical judgment. *J. Cogn. Neurosci.e* 19 684–693. 10.1162/jocn.2007.19.4.684 17381258

[B155] Simon H. A. (1956). Rational choice and the structure of the environment. *Psychol. Review* 63:129. 10.1037/h0042769 13310708

[B156] Simon H. A. (1990). Invariants of human behavior. *Annu. Rev. Psychol.* 41 1–20. 10.1146/annurev.ps.41.020190.000245 18331187

[B157] Solé R. V. Ferrer-Cancho R. Montoya J. M. Valverde S. (2002). Selection, tinkering, and emergence in complex networks. *Complexity* 8 20–33. 10.1002/cplx.10055

[B158] Spellman B. A. Schnall S. (2009). Embodied rationality. *Queens Law J.* 35 116–117. 10.2139/ssrn.1404020 34566788

[B159] Stanovich K. (2011). *Rationality and the Reflective Mind.* Oxford: Oxford University. 10.1093/acprof:oso/9780195341140.001.0001

[B160] Sterelny K. (2007). Social intelligence, human intelligence and niche construction. *Philos. Trans. R. Soc. B Biol. Sci.* 362 719–730. 10.1098/rstb.2006.2006 17255005 PMC2346527

[B161] Sterelny K. (2010). Minds: extended or scaffolded? *Phenomenol. Cogn. Sci.* 9 465–481. 10.1007/s11097-010-9174-y

[B162] Todd P. M. Gigerenzer G. (2000). Précis of” Simple heuristics that make us smart”. *Behav. Brain Sci.* 23 727–741. 10.1017/S0140525X00003447 11301545

[B163] Tooby J. Cosmides L. (1992). “The psychological foundations of culture,” in *The Adapted Mind: Evolutionary Psychology and the Generation of Culture*, eds Barkow J. Cosmides L. Tooby J. (New York, NY: Oxford University Press), 19–136.

[B164] Tooby J. Cosmides L. (2005). “Conceptual foundations of evolutionary psychology,” in *The Handbook of Evolutionary Psychology*, ed. Buss D. (Hoboken, NJ: Wiley), 5–67. 10.1002/9780470939376.ch1

[B165] Tooby J. Cosmides L. (2007). Evolutionary psychology, ecological rationality, and the unification of the behavioral sciences. *Behav. Brain Sci.* 30 42–43. 10.1017/S0140525X07000854

[B166] Tramacere A. Ferrari P. F. (2016). Faces in the mirror, from the neuroscience of mimicry to the emergence of mentalizing. *J. Anthropol. Sci.* 94 1–14. 27176898 10.4436/JASS.94037

[B167] Tramacere A. Pievani T. Ferrari P. F. (2017). Mirror neurons in the tree of life: mosaic evolution, plasticity and exaptation of sensorimotor matching responses. *Biol. Rev.* 92, 1819–1841. 10.1111/brv.12310 27862868 PMC5433930

[B168] Tversky A. Kahneman D. (1974). Judgment under uncertainty: heuristics and biases. *Science* 185 1124–1131. 10.1126/science.185.4157.1124 17835457

[B169] Tversky A. Kahneman D. (1983). Extension versus intuitive reasoning: the conjunction fallacy in probability judgment. *Psychol. Rev.* 90 293–315. 10.1037/0033-295X.90.4.293 26635712

[B170] Uchiyama R. Spicer R. Muthukrishna M. (2020). Cultural evolution of genetic heritability. *Behav. Brain Sci.* 21 1–147. 10.1101/2020.06.23.167676 34016199

[B171] Vera A. H. Simon H. A. (1993). Situated action: a symbolic interpretation. *Cogn. Sci.* 17 7–48. 10.1207/s15516709cog1701_2

[B172] Vrba E. S. Gould S. J. (1986). The hierarchical expansion of sorting and selection: sorting and selection cannot be equated. *Paleobiology* 12 217–228. 10.1017/S0094837300013671

[B173] Walsh V. (2003). A theory of magnitude: common cortical metrics of time, space and quantity. *Trends Cogn. Sci.* 7 483–488. 10.1016/j.tics.2003.09.002 14585444

[B174] Williams G. C. (1966). *Adaptation and Natural Selection: A Critique of Some Current Evolutionary Thought.* Princeton, NJ: Princeton University Press.

[B175] Williams G. C. (1992). Gaia, nature worship and biocentric fallacies. *Q. Rev. Biol.* 67 479–486. 10.1086/417796

[B176] Wilson M. (2002). Six views of embodied cognition. *Psychonom. Bull. Rev.* 9 625–636. 10.3758/BF03196322 12613670

[B177] Wittgenstein L. (1953). *Philosophical Investigations.* New York, NY: Macmillan Publishing Company.

